# Supplementary material for: Indoxyl Sulfate Stimulates Angiogenesis by Regulating Reactive Oxygen Species Production via CYP1B1
Source: Toxins (Basel). 2019 Aug 2;11(8):454. doi: 10.3390/toxins11080454 (PMC6723868; doi:10.3390/toxins11080454)
Supplement: Supplementary file 1 [file toxins-11-00454-s001.zip › Supplementary Figure 1.pdf]

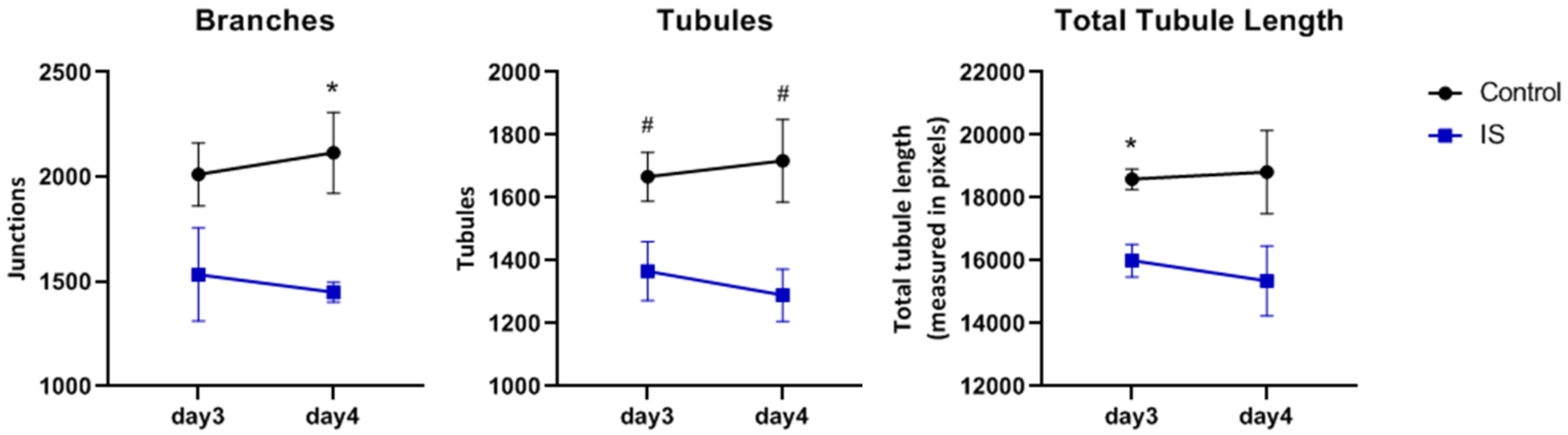

Supplementary Figure 1. IS-affected angiogenic responses compared to the control group with no IS stimulation at day 3 and day 4 respectively in a 3D co-culture model, in CYP1B1 silenced HUVECs. Without CYP1B1 expression, the number of junctions, the number of tubules and the total tubule length were lower in IS group than in the control group at both day 3 and day 4 (n=3). A reduction was observed in all three parameters from day 3 to day 4 under IS stimulation. In contrast, in the absence of IS, all three parameters remained stable between day 3 and day 4. All values are presented in mean ± SEM, t-test was performed between IS and control group per time-point, #p-value<0.1, *p-value<0.05.
