# Supplementary material for: Indoxyl Sulfate Stimulates Angiogenesis by Regulating Reactive Oxygen Species Production via CYP1B1
Source: Toxins (Basel). 2019 Aug 2;11(8):454. doi: 10.3390/toxins11080454 (PMC6723868; doi:10.3390/toxins11080454)
Supplement: Supplementary file 1 [file toxins-11-00454-s001.zip › Supplementary Figure 2.pdf]

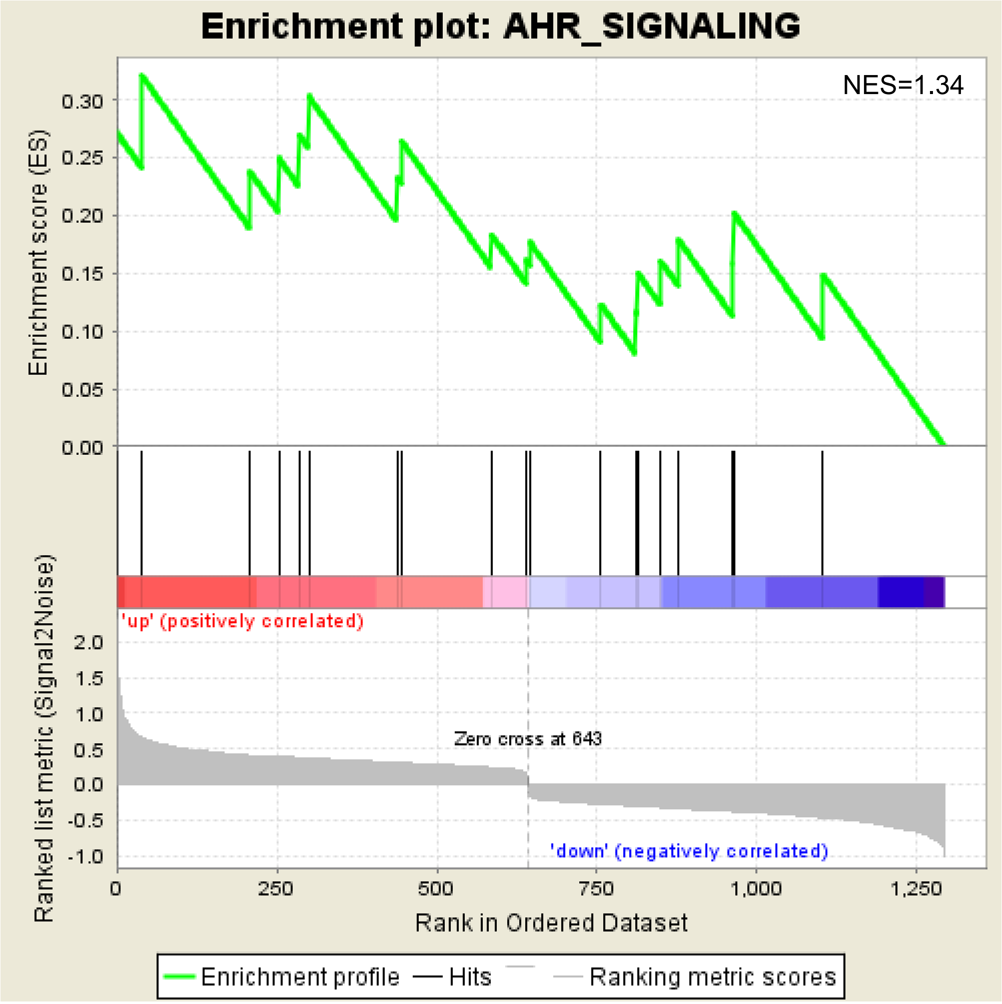

Supplementary Figure 2. Gene set enrichment plot for the AhR signalling. Significant differentially expressed genes from our study are ranked based on their fold change and shown in the bottom part. The running statistics of the AhR gene set throughout the ranked list of genes is presented by the green line in the upper part and the enrichment score is shown on the y-axis. The position of each overlapping gene between AhR gene set and the expression dataset were indicated by the black bar in the middle part. NES: normalized enrichment score.
