# Supplementary material for: Indoxyl Sulfate Stimulates Angiogenesis by Regulating Reactive Oxygen Species Production via CYP1B1
Source: Toxins (Basel). 2019 Aug 2;11(8):454. doi: 10.3390/toxins11080454 (PMC6723868; doi:10.3390/toxins11080454)
Supplement: Supplementary file 1 [file toxins-11-00454-s001.zip › Supplementary Report 1.pdf]

GSEA Report for Dataset Differentially_expressed_genes_RNAseq

Enrichment in phenotype: up (1 samples)

- 1 / 1 gene sets are upregulated in phenotype **up**
- 1 gene sets are significant at FDR < 25%
- 0 gene sets are significantly enriched at nominal pvalue < 1%
- 0 gene sets are significantly enriched at nominal pvalue < 5%
- Snapshot of enrichment results
- Detailed enrichment results in html format
- Detailed enrichment results in excel format (tab delimited text)
- Guide to interpret results

Enrichment in phenotype: down (1 samples)

- None of the gene sets are enriched in phenotype **down**
- Guide to interpret results

Dataset details

- The dataset has 1293 features (genes)
- No probe set => gene symbol collapsing was requested, so all 1293 features were used

Gene set details

- Gene set size filters (min=15, max=500) resulted in filtering out 0 / 1 gene sets
- The remaining 1 gene sets were used in the analysis
- List of gene sets used and their sizes (restricted to features in the specified dataset)

Gene markers for the up *versus* down comparison

- The dataset has 1293 features (genes)
- # of markers for phenotype **up**: 643 (49.7%) with correlation area 49.4%
- # of markers for phenotype **down**: 650 (50.3%) with correlation area 50.6%

- Detailed rank ordered gene list for all features in the dataset
- Heat map and gene list correlation profile for all features in the dataset

Global statistics and plots

- Plot of p-values *vs.* NES
- Global ES histogram

Other

- Parameters used for this analysis

Comments

- Timestamp used as random seed: 1562578785087

---

xtools.gsea.Gsea [Mon, Jul 8, '19 11 AM 39] Report: AhR_enrichment.Gsea.1562578785071.rpt by user: Jiayi Website: www.gsea-msigdb.org/gsea Questions & Suggestions: Contact page
